# p16-3MR: A Novel Model to Study Cellular Senescence in Cigarette Smoke-Induced Lung Injuries

**DOI:** 10.3390/ijms22094834

**Published:** 2021-05-03

**Authors:** Gagandeep Kaur, Isaac K. Sundar, Irfan Rahman

**Affiliations:** 1 Department of Environmental Medicine, University of Rochester Medical Center, Rochester, NY 14642, USA; gagandeep_kaur@urmc.rochester.edu; 2 Department of Internal Medicine, Division of Pulmonary Critical Care and Sleep Medicine, University of Kansas Medical Center, Kansas City, KS 66160, USA; isundar@kumc.edu

**Keywords:** p16, mitochondrial dysfunction, cellular senescence, SASP, cigarette smoke, COPD

## Abstract

Cellular senescence and lung aging are associated with the pathogenesis of chronic obstructive pulmonary disease (COPD). COPD progresses with aging, and chronic smoking is the key susceptibility factor in lung pathological changes concurrent with mitochondrial dysfunction and biological aging. However, these processes involving cigarette smoke (CS)-mediated lung cellular senescence are difficult to distinguish. One of the impediments to studying cellular senescence in relation to age-related lung pathologies is the lack of a suitable in vivo model. In view of this, we provide evidence that supports the suitability of p16-3MR mice to studying cellular senescence in CS-mediated and age-related lung pathologies. p16-3MR mice have a trimodal reporter fused to the promoter of the p16^INK4a^ gene that enables detection, isolation, and selective elimination of senescent cells, thus making them a suitable model to study cellular senescence. To determine their suitability in CS-mediated lung pathologies, we exposed young (12–14 months) and old (17–20 months) p16-3MR mice to 30 day CS exposure and studied the expression of senescent genes (p16, p21, and p53) and SASP-associated markers (MMP9, MMP12, PAI-1, and FN-1) in air- and CS-exposed mouse lungs. Our results showed that this model could detect cellular senescence using luminescence and isolate cells undergoing senescence with the help of tissue fluorescence in CS-exposed young and old mice. Our results from the expression of senescence markers and SASP-associated genes in CS-exposed young and old p16-3MR mice were comparable with increased lung cellular senescence and SASP in COPD. We further showed alteration in the; (i) tissue luminescence and fluorescence, (ii) mRNA and protein expressions of senescent markers and SASP genes, and (iii) SA-β-gal activity in CS-exposed young and old p16-3MR mice as compared to their air controls. Overall, we showed that p16-3MR is a competent model for studying the cellular senescence in CS-induced pathologies. Hence, the p16-3MR reporter mouse model may be used as a novel tool for understanding the pathobiology of cellular senescence and other underlying mechanisms involved in COPD and fibrosis.

## 1. Introduction

Cigarette smoke (CS) can cause DNA damage and cellular senescence, leading to premature lung aging [1,2,3,4]. Reports prove a critical role of cigarette smoke-induced cellular senescence in the development of chronic obstructive pulmonary disease (COPD) or emphysema [3,5,6,7]. Markers of senescence, including p16 and p21, have been shown to be upregulated in both the airway epithelium and endothelium of lung specimens from patients with COPD, thus proving that cellular senescence has an important role in the pathophysiology of COPD [3,6,8]. Cellular senescence refers to the state of irreversible cell cycle arrest in somatic cells in response to intrinsic stressors (DNA damage) or extrinsic stressors (oxidative stress) [9]. Unlike cells that have undergone apoptosis, senesced cells remain metabolically active and continue to affect their surrounding cells after having undergone specific phenotypic changes themselves. Among these phenotypic changes are an increased production of extracellular vesicles, mitochondrial dysfunction, and an increased secretion of inflammatory cytokines and interleukins. Changes in the secretory profile of a senescent cell reshape not only its microenvironment, but also that of its surrounding cells, and are termed as senescence-associated secretory phenotype (SASP) [9,10]. SASP has been shown to be linked to chronic inflammation, which is a ubiquitous component of aging tissues and most age-related diseases including COPD and idiopathic pulmonary fibrosis (IPF) [10,11,12]. Mounting evidence proves that the elimination of these senescent cells can prevent the development and/or exacerbation of certain age-related pathologies [13,14,15,16,17].

The major impediment in studying the role of senescent cells in age-related pathologies is the lack of a suitable reporter model. To overcome this limitation, Demaria et al. generated a novel mouse model, p16-3MR, which can (a) detect senescent cells in living animals, (b) enable the isolation of senesced cells from mouse tissues, and (c) eliminate senescent cells upon treatment with a drug otherwise ineffective in wild-type mice. p16-3MR mice carry a transgene consisting of a trimodal reporter under the regulation of a p16 promoter [18]. p16 is a tumor suppressor gene involved in regulating cellular senescence, specifically through its induction of G1 cell cycle arrest by inhibiting cyclin-dependent kinases [3,19]. Theoretically, p16-3MR is a useful model for studying cellular senescence in various age-related pathologies [15,16,17]; however, to our knowledge, the efficacy of this model system in studying lung pathologies induced by cigarette smoke exposure has not been tested.

In light of this, we studied the suitability of using p16-3MR as a model to study cellular senescence in cigarette smoke-induced pulmonary pathologies. Previous reports by our group have shown that chronic CS exposure induced the upregulation of cellular senescence as well as the expression of p16 in C57BL/6J mice, independent of age [20]. In this study, we determined whether p16-3MR reporter mice could be used in studying the role of lung cellular senescence in the pathophysiology of COPD. We analyzed the changes in the expression of SASP markers following 30 days CS exposure in both young and old p16-3MR mice. Using In Vivo Imaging System (IVIS) and fluorescence microscopy of lung tissues, we show that p16-3MR mice can be successfully used to visualize and isolate senescent cells from the lungs of CS-exposed mice.

## 2. Results

### 2.1. Sub-Chronic CS Exposure Augments Luminescence Indicative of p16 Expression in the Lungs of p16-3MR Reporter Mice

**Figure 1:**
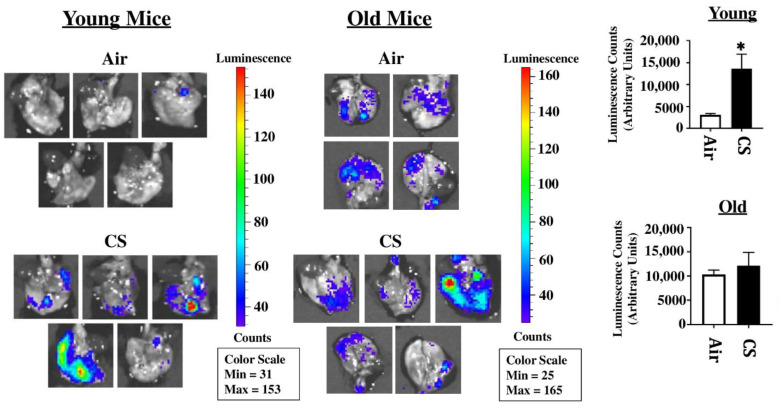
Detection of cellular senescence using tissue luminescence in the lungs of CS-exposed p16-3MR mice. Young and old p16-3MR mice were subjected to sub-chronic (30 days) CS exposure. The lung tissues from air- and CS-exposed mice were harvested and the lung tissue luminescence was measured using an IVIS^®^ Spectrum multispectral imaging instrument. Data was normalized using the tissue luminescence from the lung tissues of wild-type (C57BL/6J) mice. Representative images of tissue luminescence from each sample were provided and the quantified luminescence counts from each sample plotted alongside. Data are shown as mean ± SEM (*n* = 4–5/group). * *p* < 0.05; vs. air as per unpaired *t*-test.

p16-3MR mice are a unique model designed to study cellular senescence by the fusion of a senescence-sensitive promoter of p16^INK4a^ and its adjacent gene p19^Arf^ with a trimodal reporter constituted of functional domains for LUC (Renilla Luciferase), mRFP (monomeric red fluorescent protein), and HSV-TK (truncated herpes simplex virus 1 thymidine kinase). We tested the suitability of this model to study CS-induced cellular senescence by exposing young and old p16-3MR mice to sub-chronic CS and harvesting the lung tissues. The 3MR-expressing cells were first detected using luminescence in the lung tissues from air- and CS-exposed young and old mice. Our results demonstrated a significant increase in lung tissue luminescence following sub-chronic CS exposure (30 days) in young p16-3MR mice as compared to their older counterparts. However, the augmentation in cellular senescence, as indicated by the increase in tissue luminescence, was not significant in old p16-3MR mice following the 30 days CS exposure (Figure 1).

### 2.2. Sub-Chronic Exposure to CS Results in a Significant Increase in the mRFP Expression in the Lungs of Young p16-3MR Mice

**Figure 2:**
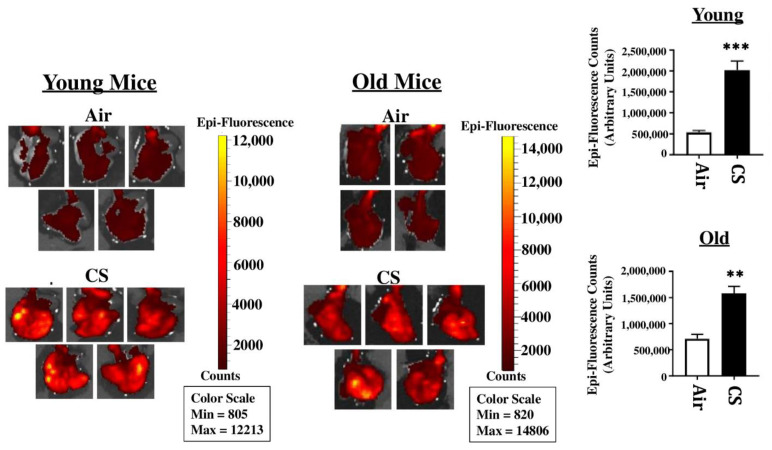
Tissue fluorescence in the lungs of CS-exposed young and old p16-3MR mice using IVIS imaging. Young and old p16-3MR mice were subjected to sub-chronic (30 days) CS exposure. The lung tissues from air- and CS-exposed mice were harvested and the lung tissue fluorescence was measured using an IVIS^®^ Spectrum multispectral imaging instrument at excitation and emission maxima of 535 and 580 nm respectively. Data was normalized using tissue fluorescence from the lung tissues of wild-type (C57BL/6J) mice. Representative images of *n* = 4–5/group were provided and the quantified fluorescence counts plotted as mean ± SEM. ** *p* < 0.01, *** *p* < 0.001; vs. air as per unpaired *t*-test.

**Figure 3:**
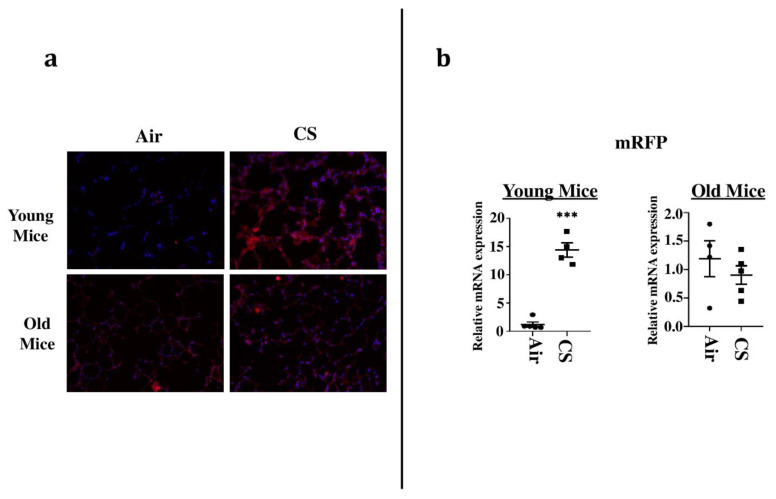
Senescent cells observed in lung tissues from CS-exposed p16-3MR mice by fluorescence microscopy. (**a**) Lung tissue sections from air- and CS-exposed younger and older p16-3MR mice were stained with DAPI and tissue fluorescence (mRFP) was observed using Nikon Eclipse Ni-U microscope. Representative images of *n* = 4–5/group were provided. Original magnification, 200×. (**b**) mRNA expression of mRFP in the lung tissue was examined by qPCR. Bar graphs represent the mean normalized fold change of respective air- vs. CS-exposed mouse lungs using ∆∆Ct method. Data are shown as mean + SEM (*n* = 4–5/group). *** *p* < 0.001, vs. air as per unpaired *t*-test.

To sort the tissues/cells undergoing cellular senescence, we tested the mRFP fluorescence and expression in the lung tissues from both CS-exposed young and old p16-3MR mice. We first employed IVIS imaging to measure the mRFP fluorescence in air- and CS-exposed lungs from younger and older groups of p16-3MR mice. Our results showed a significant increase in the lung tissue fluorescence (mRFP) following CS-exposure in both age groups, but we observed a lot of background signal in our samples (Figure 2). We suspect that this autofluorescence was due to the possibility of CS-induced tar deposition in the lung during sub-chronic CS exposure. Thus, we measured the tissue fluorescence in lung tissue sections from air/CS-exposed young and old p16-3MR mice. Our investigations showed a pronounced upregulation in mRFP expression of CS-exposed young p16-3MR mice relative to their respective age-related air controls. Contrarily, the increase in the mRFP expression in the lungs of CS-exposed old p16-3MR was not significant (Figure 3a). Furthermore, mRNA expression analyses for mRFP expression correlated with our previous findings, further substantiating our results (Figure 3b).

### 2.3. Increased Expression of Cellular-Senescence Markers (p16 and p21) Following Sub-Chronic CS Exposure in Young p16-3MR Mice

**Figure 4:**
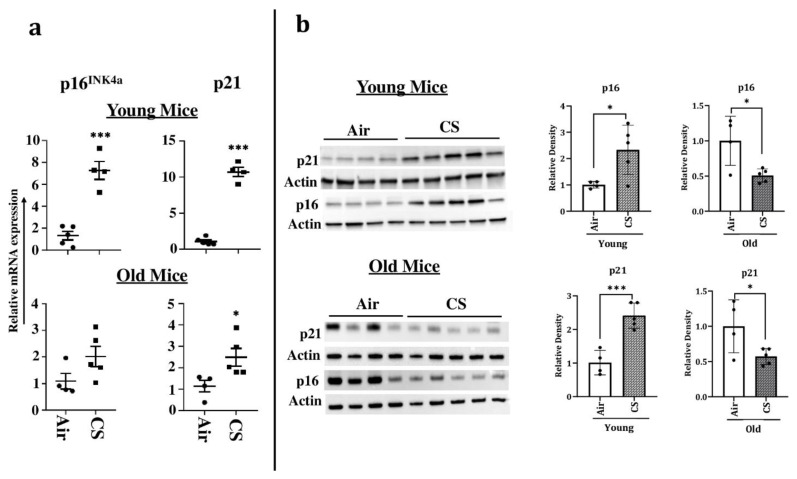
Increased mRNA and protein expression of senescence markers in CS-exposed p16-3MR mice. Younger and older mice were exposed to sub-chronic CS for 30 days. (**a**) mRNA and (**b**) protein expressions of early- (p21) and late-stage (p16) senescence markers were measured in the lung homogenates using qPCR and immunoblotting analyses respectively. β-actin- was used as loading controls. The band intensity was measured by densitometry and data were shown as fold change relative to respective control group. Full gels/blots with bands (unedited/uncropped electrophoretic gels/blots) obtained from air- and CS-exposed younger and older mouse samples from the same experiments were processed in parallel and are shown (see Supplementary Materials as Suppl. Figures file). Data are shown as mean ± SEM (*n* = 4–5/group). * *p* < 0.05, *** *p* < 0.001 vs. Air as per unpaired *t*-test.

To confirm our findings, we next determined the expression of the markers of cellular senescence p16 and p21 in our study model at both the transcriptional and translational levels. The mRNA levels of senescence markers p16^INK4a^ and p21 were significantly upregulated in young CS-exposed p16-3MR mice, confirming the presence of senescent cells following sub-chronic CS exposure (Figure 4a). Immunoblotting results supported the findings from the aforementioned mRNA expression, further substantiating our claims (Figure 4b). Contrarily, we did not find a significant increase in the mRNA or protein expression of p16^INK4a^ in older CS-exposed mice as compared to their age-related air controls (Figure 4a,b). In fact, the protein expression of p16 was significantly reduced on CS exposure in younger p16-3MR mice (Figure 4b). Similarly, though we found a significant increase in the mRNA expression of p21, its protein expression in the lung homogenates demonstrated marked reduction in CS-exposed old p16-3MR mice (Figure 4a,b).

### 2.4. Sub-Chronic CS Exposure Contributes towards the Augmentation of p53 Protein Expression and SA-β-gal Activity in the Lungs of p16-3MR Mice

**Figure 5:**
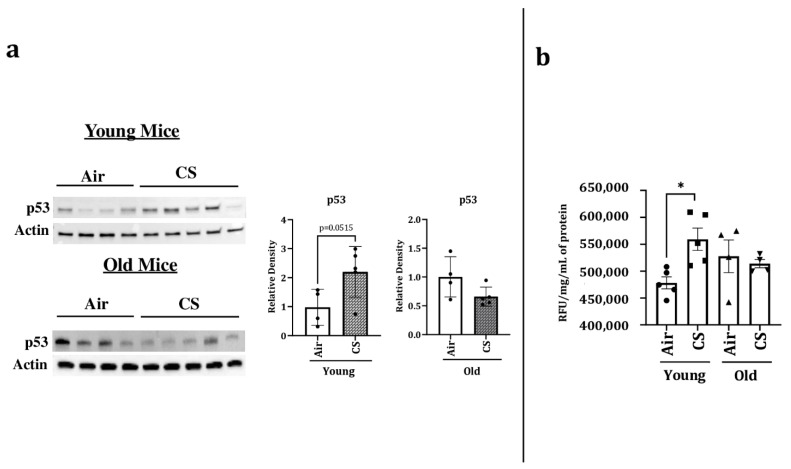
Increased levels of p53 and SA-β-gal activity in young CS-exposed p16-3MR mice. Younger and older p16-3MR mice were exposed to sub-chronic CS for 30 days, and lung homogenate was used to determine: (**a**) p53 protein expression using immunoblotting. The band intensity was measured by densitometry and data were shown as fold change relative to respective control group. Full gels/blots with bands (unedited/uncropped electrophoretic gels/blots) obtained from air- and CS-exposed younger and older mouse samples from the same experiments were processed in parallel and are shown (see Supplementary Materials as Suppl. Figures file). p-value calculated per unpaired *t*-test, and (**b**) SA-β-gal activity. Data are shown as mean ± SEM (*n* = 4–5/group). * *p* < 0.05, vs. air as per one-way ANOVA for multiple comparisons.

p53 is a transcription factor that has been linked with cellular senescence and aging. It plays a critical role in cellular response towards stress. We thus studied the protein expression of p53 in our experimental groups and observed a significant increase in the expression of p53 in CS-exposed young p16-3MR mice. However, we did not observe any change in the protein expression of p53 in air- or CS-exposed older p16-3MR mice (Figure 5a).

Considering that SA-β-gal activity (lysosomal) is a marker of cellular senescence, we measured the SA-β-gal activity in the lung homogenates of air- and CS-exposed younger and older p16-3MR mice. We observed an age-independent augmentation of SA-β-gal activity in the CS-exposed young and old mice (Figure 5b). Collectively, these results show that the characteristic of aging itself is involved in the lung cellular senescence program/process but does not increase the susceptibility of CS-induced cellular senescence in lung aging using a mouse model of COPD.

### 2.5. Alterations in the mRNA and Protein Expression of SASP-Associated Genes in CS-Exposed p16-3MR Mice

**Figure 6:**
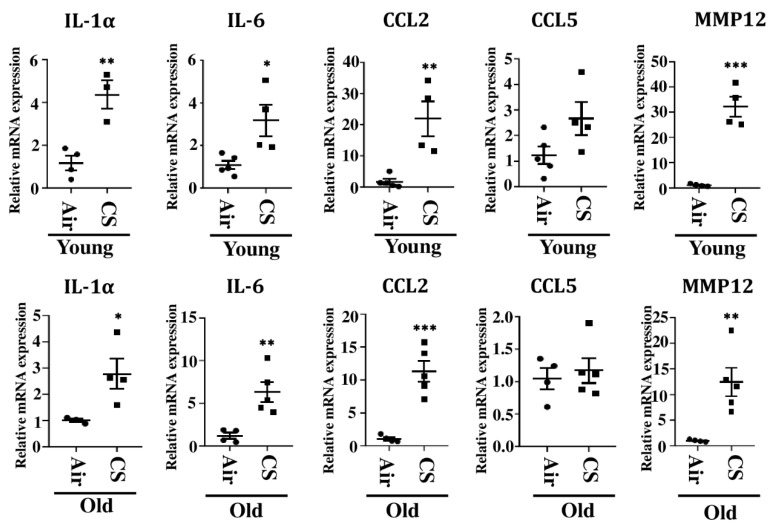
Increased mRNA expression of SASP markers in CS-exposed p16-3MR mice. mRNA expression of SASP markers (IL-1α, IL6, CCL2, CCL5 and MMP12) were measured in the lung tissues from air- and CS-exposed young and old p16-3MR mice. Scatter plot represent the mean normalized fold change of respective air- vs. CS-exposed mouse lungs using ∆∆Ct method. Data are shown as mean ± SEM (*n* = 3–5/group). * *p* < 0.05, ** *p* < 0.01, *** *p* < 0.001 vs. air as per unpaired *t*-test.

**Figure 7:**
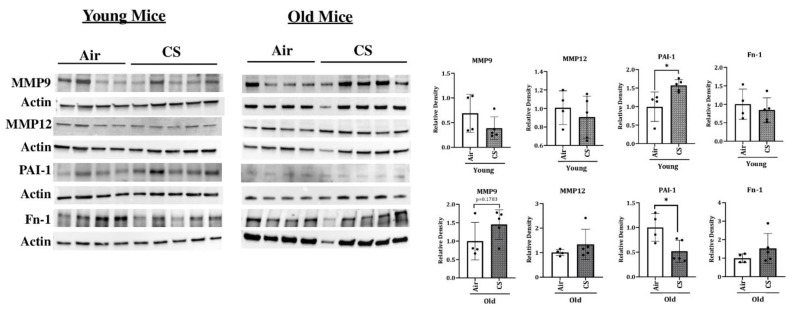
Altered protein abundance of SASP-associated markers in CS-exposed p16-3MR mice. Younger and older p16-3MR mice were exposed to sub-chronic CS for 30 days and the expression of SASP-associated markers-MMP9, MMP12, PAI-1 and FN-1-were determined using immunoblotting analyses. β-actin was used as loading controls. The band intensity was measured by densitometry and data were shown as fold change relative to respective control group. Full gels/blots with bands (unedited/uncropped electrophoretic gels/blots) obtained from air- and CS-exposed younger and older mouse samples from the same experiments were processed in parallel and are shown (see Supplementary Materials as Suppl. Figures files). Data are shown as mean ± SEM (*n* = 4–5/group). * *p* < 0.05 vs. air as per unpaired *t*-test.

To determine the changes in the protein expression of various SASP-associated proteins, we studied the expression of MMP12, MMP9, FN-1, PAI-1, and p53 in air- and CS-exposed younger and older p16-3MR mice. Consistent with the gene expression studies, we found a significant increase in the mRNA expression of MMP12 in CS-exposed young and old p16-3MR mice (Figure 6). However, the protein expression data did not show any notable variation in the expression of MMP12 in the lung of air- or CS-exposed young and old p16-3MR groups. In addition, no significant change in the expression of MMP9 was observed in response to sub-chronic CS exposure in young and old groups of p16-3MR mice, thus suggesting no changes in the extracellular matrix (ECM) composition on CS (30 days) exposure (Figure 7).

We further demonstrated a pronounced upregulation in the expression of PAI-1, a marker for lung cell senescence, in CS-exposed young p16-3MR mice (Figure 6). On the other hand, the protein expression of PAI-1 was significantly decreased in sub-chronic CS-exposed old p16-3MR mice. Contrary to this, the expression of FN-1 was not affected in 30 days CS-exposed young and old p16-3MR mice (Figure 6). We also studied the expression of inflammatory subunits of NF-κB (p50/p105) in sub-chronic CS-exposed young and old p16-3MR mice. We did not observe any significant changes in the expression of p50 or p105 in the 30 days CS-exposed p16-3MR mouse model (Supplementary Figure S1).

We further studied the mRNA expression of other SASP-related genes, IL-1α, CCL2, IL-6, and CCL5, in CS-exposed young and old p16-3MR mice. Our results demonstrated a significant increase in the mRNA expression of IL-1α, CCL2, and IL-6 in CS-exposed young and old p16-3MR mice as compared to their age-related air controls. The mRNA expression of CCL5 remained unchanged in air- and CS-exposed young and old p16-3MR mice (Figure 7).

**Figure 8:**
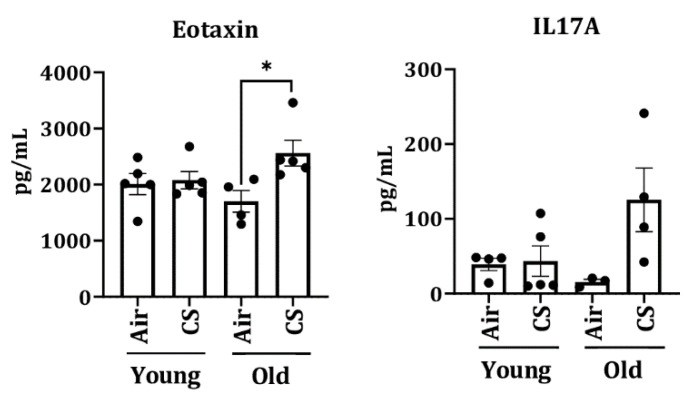
Increased level of plasma cytokines in CS-exposed p16-3MR mice. Younger and older p16-3MR mice were exposed to sub-chronic CS for 30 days and plasma was used to determine SASP cytokines. The levels of pro-inflammatory plasma cytokines (eotaxin and IL-17A) were measured using Luminex multiplex assay. Data are shown as mean ± SEM (*n* = 4–5/group). * *p* < 0.05 vs. air as per one-way ANOVA for multiple comparisons.

To further assess the senescence-mediated induction of pro-inflammatory markers on CS exposure (30 days), we measured the levels of pro-inflammatory cytokines/chemokines in the plasma of air- and CS-exposed young and old p16-3MR mice using Luminex multiplex assay. Interestingly, we observed a significant increase in the levels of eotaxin in CS-exposed older p16-3MR mice (Figure 8).

## 3. Discussion

Cellular senescence is a complex response towards stress where originally proliferating cells lose their power to proliferate. It is known to play an important role in two disparate processes, tumorigenesis and aging. Mounting evidence suggests that the senescence-induced growth arrest acts as a barrier towards tumor progression. However, accumulation of senescent cells can also drive aging-associated pathologies. Therefore, cellular senescence is the prime example of evolutionarily antagonistic pleiotropic response, which is beneficial at young age but deleterious at older ages [9,18,21].

Many cigarette smoke (CS)-related pathologies show exacerbating symptoms at older age [22,23,24]. Reports suggest that cellular senescence is responsible for aggravating disease symptoms in pathologies like asthma, COPD, and pulmonary fibrosis [6,25,26,27]. In fact, cigarette smoke has been shown to cause DNA damage, leading to premature and accelerated lung aging that ultimately leads to the development of COPD [27,28]. However, the exact role of senescence in development of these lung pathologies is not completely understood. One of the hindrances in this regard is the lack of a suitable reporter model to study lung cellular senescence, though several models are proposed that have their own pros and cons [19,29,30,31,32]. To overcome this hindrance, Demaria et al. (2014) generated a mouse model named p16-3MR that was shown to effectively identify, isolate, and selectively eliminate senescent cells [18].

In this reporter mouse model, the senescence-sensitive promoter of p16^INK4a^ and its adjacent p19^Arf^ genes were inactivated and integrated in the BAC (bacteria artificial chromosome) with a 3MR transgene. This 3MR transgene encoded three fusion proteins- luciferase (LUC), monomeric red fluorescent protein (mRFP), and herpes simplex virus thymidine kinase (HSV-TK)- allowing identification, sorting, and selective elimination of senescent cells, respectively [18]. In the current study, we assessed the suitability of this model to study lung cellular senescence in cigarette smoke-related pathologies. We exposed young (12–14 months) and old (17–20 months) p16-3MR mice to sub-chronic CS for the duration of 30 days. Thereafter, we assessed the expression of senescent markers and SASP-related genes in the lungs of control and treated groups.

On studying the tissue luminescence in lung tissues using IVIS imaging, we found a CS-induced upregulation of p16 expression indicative of increase in tissue luminescence that was significant amongst young mice. Considering that luminescence indicates cellular senescence in this model, we were effectively able to show premature induction of senescence in CS-exposed young mice in our study (Figure 1).

We next studied the mRFP expression in lung tissue sections using fluorescence microscopy to track the senescent cells (Figure 2 and Figure 3). Our results further substantiated our previous findings, thus showing that this model could effectively be used to track the CS-induced senescent cell using fluorescence. It is pertinent to mention here that we were unable to use tissue fluorescence to identify mRFP expression in the lung tissues from air- and CS-exposed young and old p16-3MR mice. IVIS imaging results showed excessive fluorescence in the whole-lung for the CS-exposed group, which could be an outcome of autofluorescence due to tar deposition on CS inhalation in these animals. We thus demonstrated that IVIS imaging cannot be used to study senescence-induced tissue fluorescence in p16-3MR mice (Figure 2). However, our fluorescence microscopy and qPCR results were able to identify CS-induced senescence in our mouse model (Figure 3). In fact, future studies could use immunohistochemical staining to identify the specific cell types undergoing cellular senescence following chronic CS exposure.

We further confirmed CS-induced senescence by increased (a) transcript levels (mRNA) and protein expression of senescent markers p16 and p21 (Figure 4), (b) mRNA levels of SASP factors- IL-1α, IL-6, CCL2 and MMP12 (Figure 7), and (c) SA-β-gal activity assay in CS-exposed group as compared to air controls (Figure 5b). Our results are comparable to our previous studies [20], thus proving that the results obtained using this model are translatable.

Since cigarette smoke-induced emphysema results in tissue remodeling and disrupted expression of ECM proteins, we further tested the expression of matrix metalloproteinases (MMP9 and MMP12), tissue plasmin regulators (PAI-1), and ECM glycoprotein (FN-1) on 30 days CS-exposed young and old p16-3MR mice. Though not significant, our results demonstrate a noticeable increase in the MMP9 protein expression in CS-exposed old p16-3MR mice as compared to their age-matched air controls. Contrarily, we found a significant increase in the levels of plasminogen activator inhibitor (PAI)-1 in CS-exposed young p16-3MR mice as compared to air-exposed control mice. The level of PAI-1 significantly decreased in CS-exposed old p16-3MR mice (Figure 6). Dysregulated levels of MMPs and their inhibitors have been associated with abnormal tissue repair in conditions like fibrosis and asthma [33,34,35]. Thus, our results show that this model is effective in simulating conditions leading to ECM deposition and fibrinolysis on CS exposure, which are similar to those found in humans [33]. Since ours was a sub-chronic (30 days CS exposure) study, we could not find conditions leading to emphysematous lungs, but our current findings prove the efficacy of this model to mimic conditions of acute and chronic exposure to cigarette smoke in vivo.

Interestingly, we observed significant changes in the expression of many proteins associated with inflammation and senescence, such as p53 and eotaxin, on CS-exposure in our mouse model (Figure 5a and Figure 8). While some of the changes were pronounced amongst younger CS-exposed mice, the others showed substantial change at an older age. These findings show that age plays a key role in regulating the disease phenotypes in cigarette smoke-associated disorders. In fact, the role of several proteins might vary depending on the age and duration of exposure, which will be studied in the future. In the past few decades, the possibility of using senolytic drugs to combat against age-associated disorders has been considered [15,16,17,18,36]. However, it is pertinent to mention here that though we refer to our study groups as “young” and “old”, there is not much change in their actual ages, and so we refrain from making age-dependent correlations in this study. Given that this model can selectively target senescent cells by the use of Ganciclovir, it will be interesting to see how the elimination of senescenced cells affects smoking-induced lung molecular changes in vivo. We intend to determine this possibility in our upcoming studies.

As stated earlier, one of the limitations of this study was the fact that the animal group termed as “young” was actually mid-aged. Hence, we were unable to draw age-dependent correlations from this study. We intend to explore this aspect in our future work. In addition, we do not provide an elaborate analysis of the transcriptomic and proteomic markers for senescence in this work, but rather specific cellular senescence markers were analyzed. A detailed transcriptomic and proteomic study of the affected cellular senescence markers would be crucial in identifying the markers affected following chronic CS exposure.

**Figure 9:**
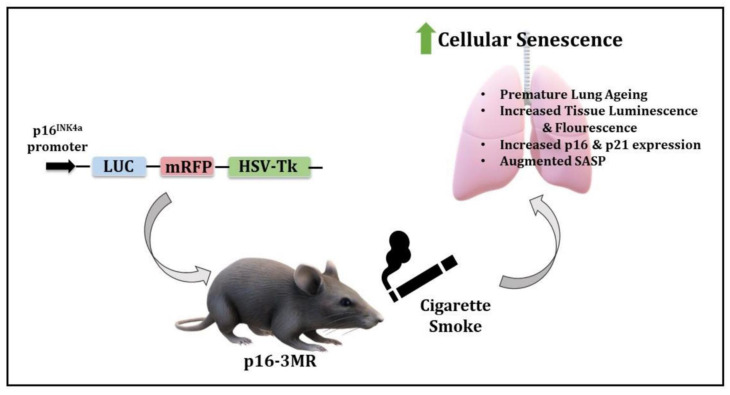
Schematics of the model and observed outcomes as shown using the p16-3MR mice exposed to cigarette smoke for cellular senescence.

Overall, we showed that the in vivo mouse model for cellular senescence using p16-3MR reporter mice could be used to study smoking-induced pathologies, such as COPD and IPF. In general, this is the first attempt to utilize p16-3MR as a senescence model in lung pathologies (Figure 9). Future studies are required with a greater focus on using the p16-3MR model to discern the role of lung cellular senescence in the progression of aging-induced conditions such as COPD, as well as to test whether the removal of senescent cells causes any alleviation in the disease phenotype. The use of this model will help to deduce the cross-talk between cellular senescence, mitochondrial dysfunction, and inflammation in smoking-associated pulmonary conditions to identify effective therapies in the future.

## 4. Materials and Methods

### 4.1. Ethics Statement and Scientific Rigor/Reproducibility

All animal experiments were performed according to the standards established by the U.S. Animal Welfare Act as per NIH guidelines. All mouse housing, handling, exposure, and procedure protocols used in this study were approved by the University Committee on Animal Research (UCAR) Committee of the University of Rochester, Rochester, NY (UCAR protocol 102204/UCAR-2007-070E, date of approval: 5 January 2019 and 3 February 2020).

Great precaution was taken in employing a robust and unbiased approach during the experimental and corresponding results analysis phases in order to ensure reproducibility befitting NIH standards. The key biological and chemical resources used in this study are of scientific standard and have been authenticated and revalidated. Unless stated otherwise, all biochemical reagents were purchased from Millipore Sigma (St. Louis, MO, USA). The antibodies listed in the study were commercial grade and were validated by their respective manufacturers.

### 4.2. p16-3MR Mouse Model

We obtained the p16-3MR mice from Dr. Judith Campisi of the Buck Institute for Research on Aging via Unity Biotechnology, Inc., San Francisco, CA, USA for conducting our experiments. p16-3MR mice are diploid for p16^INK4a^ and p19^Arf^ with a trimodal (3MR) reporter fusion protein designed to identify, isolate, and selectively kill senescent cells [18]. We used these mice upon genotyping to test the suitability of this model for studying cellular senescence in cigarette-smoke exposure-related lung pathologies. Animals that underwent cellular senescence showed an increased expression of p16. These senesced cells could be identified with luminescence and red fluorescence protein (RFP) and selectively eliminated by treatment with Ganciclovir in the p16-3MR mice model. All the mice were housed in the vivarium facility at the University of Rochester Medical Center with a 12 h light/12 h dark cycle (lights on at 6:00 a.m.). All the animals used in the study were genotyped prior to CS exposure.

### 4.3. Sub-Chronic CS Exposure

Male and female mice of different age groups (12–14 months and 17–20 months) were exposed to sub-chronic cigarette smoke generated by research grade cigarettes (3R4F) according to the Federal Trade Commission protocol (1 puff/min of 2 s duration and 35 mL volume for a total of 8 puffs at a flow rate of 1.05 L/min) with a Baumgartner-Jaeger CSM2072i automatic CS generating machine (CH Technologies, Westwood, NJ) [37,38]. The mainstream smoke exposure was performed at a concentration of ~250–300 mg/m^3^ total particulate matter (TPM) by adjusting the flow rate of the diluted medical air, and the level of carbon monoxide in the chamber measured at ~350 ppm, as described previously [37]. At the end of the exposure, 12–14 month old mice were referred to as “young,” whereas 17–20 month old mice were termed “old”. We use the same terms to denote these two groups in the rest of this manuscript. Mice that were not exposed to CS were considered the “air” group and kept in filtered air, which served as the control group in these experiments. Twenty-four hours following the final exposure, all the mice were euthanized, and their lung tissues were used for imaging, biochemical, and immunohistochemical analyses.

### 4.4. Tissue Luminescence and Fluorescence Using IVIS Imaging

To identify senescent cells using luminescence, lung tissues harvested from euthanized mice were soaked for 10 min in pre-warmed (37 °C) PBS with 2% FBS and a 1:10 dilution of Xenolight RediJect Coelenterazine h (Cat# 706506, Perkin Elmer, Waltham, MA, USA). Following a 12–15 min incubation, tissues were transferred to a fresh 35 mm dish, and luminescence was measured using the IVIS^®^ Spectrum multispectral imaging instrument (Caliper Life Sciences, Inc.—Hopkinton, MA, USA). The IVIS^®^ Spectrum multispectral imaging instrument was also used to measure the lung tissue fluorescence (RFP) in our control and experimental groups at an excitation maximum of 535 nm and emission maximum of 580 nm.

### 4.5. Fluorescence Microscopy in Lung Tissue Section

Fluorescence imaging was employed to determine mRFP expression in the lung tissue sections from CS- and air-exposed mice. More specifically, non-lavaged mouse lungs were inflated with 50% solution of optimal cutting temperature (OCT) compound and 10 μm frozen sections were cut using a rotary microtome-cryostat. Immediately after sectioning, samples were fixed and mounted using Prolong (Cat# P36962, Life technologies Corporation, Eugene, OR, USA) with DAPI. Images were acquired using a Nikon Eclipse Ni-U fluorescence microscope at 200× magnification using Advance SPOT software.

### 4.6. mRNA Expression Analyses Using qPCR

A reverse-transcriptase polymerase chain reaction (RT-PCR) was performed to determine the differential expression of cellular senescence genes in our experimental groups. Briefly, RNA was isolated from the lung tissue using the RNeasy miniprep kit (Qiagen, Valencia, CA, USA). RNA quantity and quality were assessed using a Nanodrop 1000 spectrophotometer (Thermo Fisher Scientific), and 1 μg of total RNA was used for cDNA conversion using the RT2 first strand kit (Cat# 330404, Qiagen, Valencia, CA, USA). The prepared cDNA was diluted and used to determine the expression of genes of interest using specific primers. All of the real-time PCR reactions were performed with RT2 SYBR Green/ROX PCR Master Mix (Cat# 330503, Qiagen, Valencia, CA, USA), and relative mRNA expression of each gene was determined using the CFX96 real-time system (Bio-Rad, Hercules, CA, USA). Differential expression of target genes in total RNA isolated from air- and CS-exposed lung tissues from young and old p16-3MR mice were expressed as relative fold change. As housekeeping control, 18S rRNA was used. Fold change (2^(—Delta Delta Ct)) was the normalized gene expression (2^(—Delta Ct)) in the CS-exposed group (treated) divided by the normalized gene expression (2^(—Delta Ct)) in the air group (control) [39,40]. The sequence of the primers used for amplification is provided in Supplementary Table S1.

### 4.7. Immunoblot Analysis

To determine the protein expression of various senescence-associated proteins, we employed immunoblotting. Briefly, one lobe of the lung tissue (~40 mg) was homogenized (Pro 200 homogenizer, at maximum speed, 5th gear for 40 s) in 0.3 mL of ice-cold RIPA buffer containing complete protease inhibitor cocktail (Cat# 78444, Thermo Scientific, Waltham, MA, USA). The tissue homogenate was incubated on ice for one hour to allow complete cell lysis. Following this incubation, the homogenate was centrifuged at 13,000× *g* for 30 min at 4 °C. The supernatant was aliquoted and stored at −80 °C until further analyses. A small fraction of the tissue lysate was taken and diluted for protein analysis with the help of bicinchoninic acid (BCA) colorimetric assay (Thermo Scientific, Waltham, MA, USA), where BSA was used as a standard.

Equal amounts of protein from each sample (20 μg) were resolved on a sodium dodecyl sulfate (SDS)-polyacrylamide gel and subsequently electroblotted onto nitrocellulose membranes. The nitrocellulose membranes were then blocked using 5% milk solution for one hour at room temperature. Thereafter, the membranes were probed with antibodies targeted for the protein of interest. Antibodies towards anti-p16 (Cat# sc-377412, Santa Cruz Biotechnology, Dallas, TX, USA), anti-p21 (Cat# 556430, BD Biosciences, San Jose, CA, USA), anti-Fn1 (Cat# ab2413), anti-PAI-1 (Cat# ab66705), anti-NF-κB(Cat# ab32360), anti-MMP9 (Cat# ab38898) (Abcam, Cambridge, UK), anti-MMP12 (Cat# NBP2-67344, Novus Biologicals, Littleton, CO, USA), anti-p53 (Cat# 2524), and anti-β-actin (Cat# 12620, Cell Signaling, Danvers, MA, USA) were added using a 1:1000 dilution (in 5% BSA in tris-buffered saline (TBS) containing 0.1% Tween 20) and incubated overnight at 4 °C. The following day, blots were washed three times with 1X TBST and probed with the appropriate anti-rabbit (Cat# 170-6515, Bio-Rad, Hercules, CA, USA) or anti-mouse (Cat# 7076, Cell Signaling, Danvers, MA, USA) secondary antibody (1:3000 dilution in 5% milk) for one hour. Chemiluminescence was detected through the Bio-Rad ChemiDoc MP Imaging system using the SuperSignal West Femto Maximum Sensitivity Substrate (Cat# 34096, Thermo Scientific, Waltham, MA, USA). Bio-Rad Image Lab software was used for densitometric analyses. Corresponding band intensities for the proteins of interest were plotted as fold change relative to their respective β-actin loading control bands.

### 4.8. Measurement of SA-β-gal Activity

SA-β-gal activity was measured using a cellular senescence activity assay kit (Cat# ENZ-KIT129-0120, Enzo Life sciences, Farmingdale, NY, USA) as per the manufacturer’s protocol. Briefly, one lobe of the lung tissue (~40 mg) was homogenized (Pro 200 homogenizer) in 0.3 mL of ice-cold 1X cell lysis buffer containing complete protease inhibitor cocktail (Cat# 78444, Thermo Scientific, Waltham, MA, USA). Tissue homogenate was incubated on ice for 30 min and then centrifuged at 13,000× *g* rpm for 15 min at 4 °C. The supernatant was collected and stored until further analyses. Fifty milliliters of cell lysate was mixed with 50 µL of assay buffer and incubated for 3 h at room temperature. Following incubation, 50 µL of the reaction mixture was added to 200 µL of Stop solution, and fluorescence was read using Cytation 5 (Biotek, Winooski, VA, USA) at 360 nm (Excitation)/465 nm (Emission).

### 4.9. Assessment of Pro-Inflammatory Mediators Using Luminex

The level of proinflammatory mediators in plasma (50 μL) was measured with the help of Bio-Plex Pro Mouse Cytokine Standard 23-Plex (Cat# 64209360, Bio-Rad, Hercules, CA, USA) as per manufacturer’s protocol. Blood plasma was diluted twofold, and the levels of each cytokine/chemokine were expressed as pg/mL.

### 4.10. Statistical Analysis

All statistical calculations were performed using GraphPad Prism 8.0. Data are expressed as mean ± SEM. Pairwise comparisons were done using unpaired *t*-test. For multi-group comparisons, one-way analysis of variance (ANOVA) with ad-hoc Tukey’s test was employed. All animal experiments (*n* = 4–5 mice/group) were performed twice. Differences were considered statistically significant at * *p* < 0.05, ** *p <* 0.01, and *** *p <* 0.001 when compared with respective air controls.

## Data Availability

Not applicable.

## Supplementary Materials

The following are available online at https://www.mdpi.com/article/10.3390/ijms22094834/s1.
