# Supplementary material for: p16-3MR: A Novel Model to Study Cellular Senescence in Cigarette Smoke-Induced Lung Injuries
Source: Int J Mol Sci. 2021 May 3;22(9):4834. doi: 10.3390/ijms22094834 (PMC8125702; doi:10.3390/ijms22094834)
Supplement: Supplementary file 1 [file ijms-22-04834-s001.zip › ijms-1176781-supplementary.pdf]

Supplementary Material

p16-3MR: A Novel Model to Study Cellular Senescence in Cigarette Smoke-Induced Lung Injuries

Gagandeep Kaur [1], Isaac K. Sundar [2] and Irfan Rahman [1*]

[1] Department of Environmental Medicine, University of Rochester Medical Center, Rochester, 14642 NY, USA; gagandeep_kaur@urmc.rochester.edu

[2] Department of Internal Medicine, Division of Pulmonary Critical Care and Sleep Medicine, University of Kansas Medical Center, KS 66160 Kansas City, USA; isundar@kumc.edu

* Correspondence: Irfan Rahman (IR): irfan_rahman@urmc.rochester.edu

Supplementary Table S1. Real Time-PCR primer sequences.

Gene	Primer Sequence
$p16^{INK4a}$-Fw	5′-aatctccgcgaggaaagc-3′
$p16^{INK4a}$-Rv	5′-gtctgcagcggactccat-3′
p21-Fw	5′-ttgccagcagaataaaaggtg-3′
p21-Rv	5′-tttgctcctgtgcggaac-3′
mRFP-Fw	5′-gacctcggcgtcgtagtg3′
mRFP-Rv	5′-aagggcgagatcaagatgag-3′
IL-1a-Fw	5′-tccataacccatgatctggaa-3′
IL-1a-Rv	5′-ttggttgagggaatcattcat-3′
IL-6-Fw	5′-gctaccaaactggatataatcagga-3′
IL-6-Rv	5′-ccaggtagctatggtactccagaa-3′
CCL2-Fw	5′-catccacgtgttggctca-3′
CCL2-Rv	5′-gatcatcttgctggtgaatgagt-3′
CCL5-Fw	5′-tgcagaggactctgagacagc-3′
CCL5-Rv	5′-gagtggtgtccgagccata-3′
MMP12-Fw	5′-tgaatttgcatttcctgtacatagt-3′
MMP12-Rv	5′-tgctgtaagtccatgggtga-3′
Actin-Fw	5′-ctaaggccaaccgtgaaaag-3′
Actin-Rv	5′-accagaggcatacagggaca-3′
Tubulin-Fw	5′-ctggaacccacggtcatc-3′
Tubulin-Rv	5′-gtggccacgagcatagttatt-3′

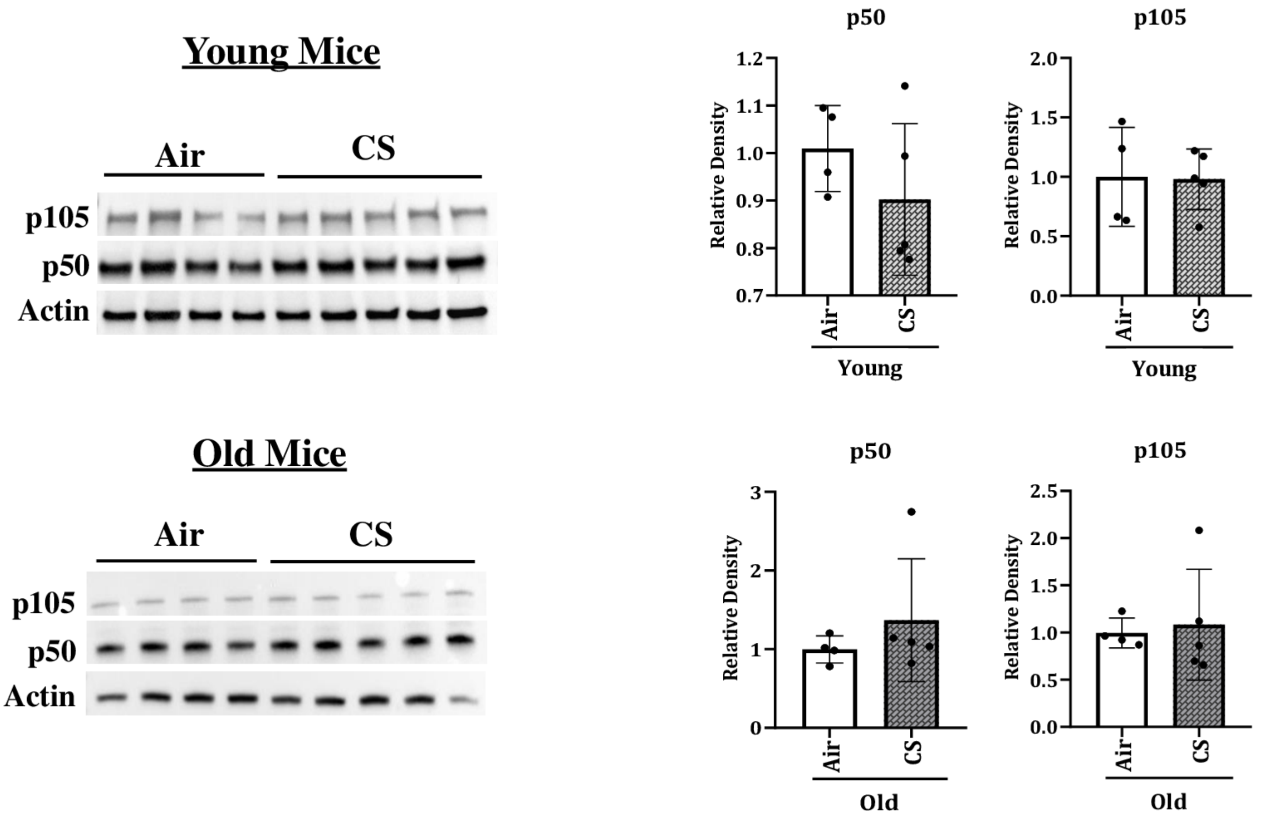

Supplementary Figure S1. Altered expression of NF-kB in CS-exposed p16-3MR mice. Younger and older p16-3MR mice were exposed to sub-chronic CS for 30 days and the expression of marker for inflammation (p50 and p105) was determined using immunoblotting analyses. β-actin was used as loading controls. The band intensity was measured by densitometry and data were shown as fold change relative to respective control group. Full Gels/blots with bands (unedited/uncropped electrophoretic gels/blots) obtained from air- and CS-exposed younger and older mouse samples from the same experiments were processed in parallel are shown (see Supplementary information as Suppl Figs. file). Data are shown as mean ± SEM (n = 4–5/group). *$p < 0.05$ vs Air as per unpaired t-test.

Uncut Original Blots

Full unedited gel for Supplementary Figure 1

p50/105 and respective β-Actin

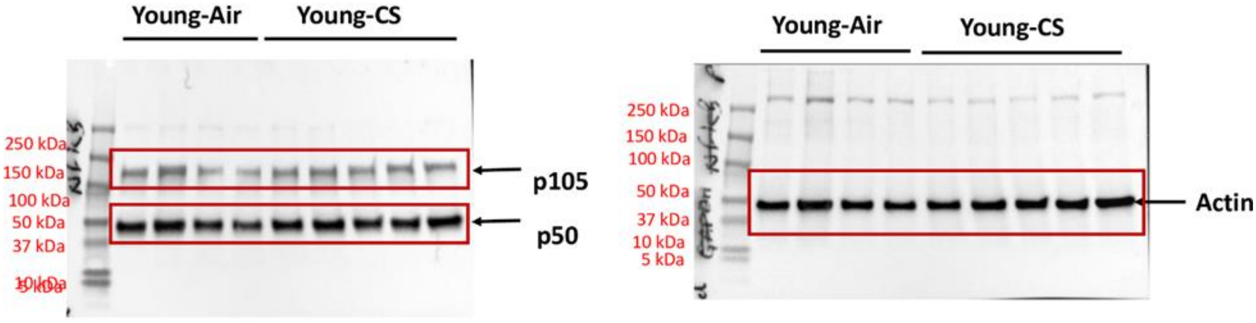

This shows the original uncut blots for p50/150 and β-actin as shown in Figure 6a of the manuscript

Uncut Original Blots

Full unedited gel for Supplementary Figure 1

p50/105 and respective β-Actin

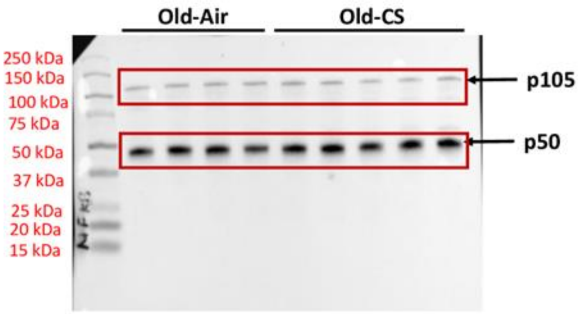

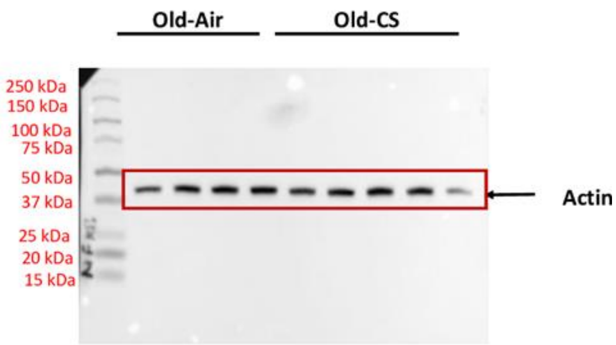

This shows the original uncut blots for p50/150 and β-actin as shown in Figure 6a of the manuscript

Uncut Original Blots

Full unedited gel for Figure 4b
p21 and respective β-Actin

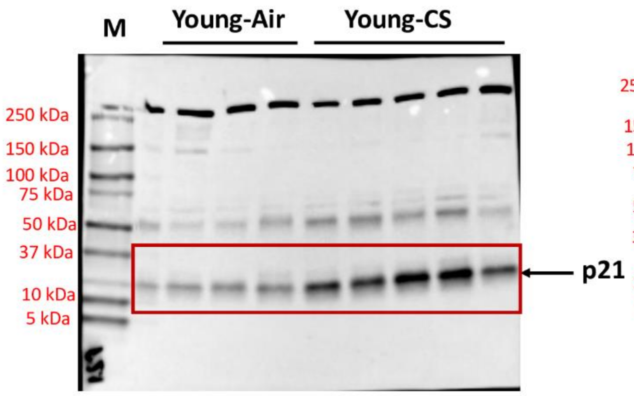

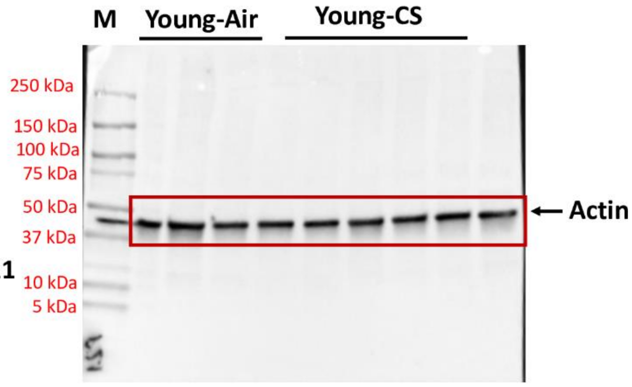

This shows the original uncut blots for p21 and β-actin as shown in Figure 4b of the manuscript

Uncut Original Blots

Full unedited gel for Figure 4b
p16 and respective β-Actin

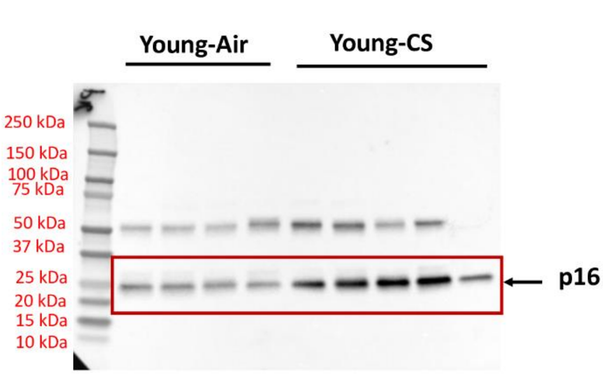

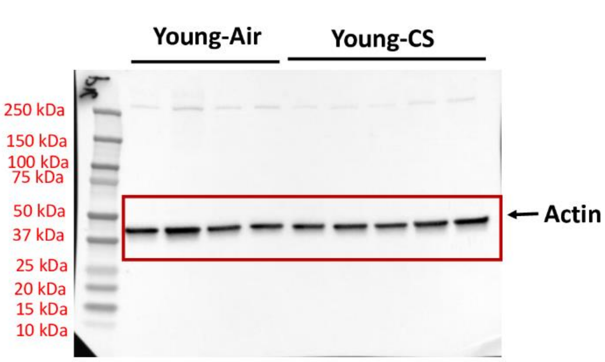

This shows the original uncut blots for p16 and β-actin as shown in Figure 4b of the manuscript

Uncut Original Blots

<u>Full unedited gel for Figure 4b</u>

p21 and respective β-Actin

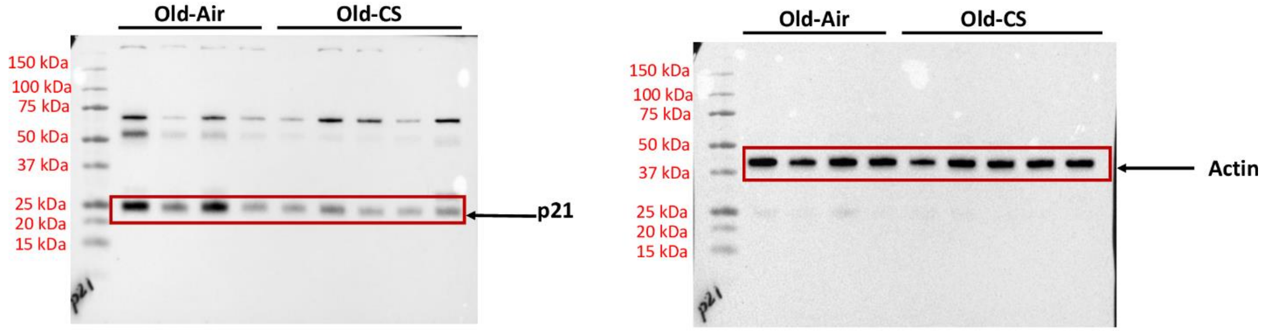

This shows the original uncut blots for p21 and β-actin as shown in Figure 4b of the manuscript

Uncut Original Blots

<u>Full unedited gel for Figure 4b</u>

p16 and respective β-Actin

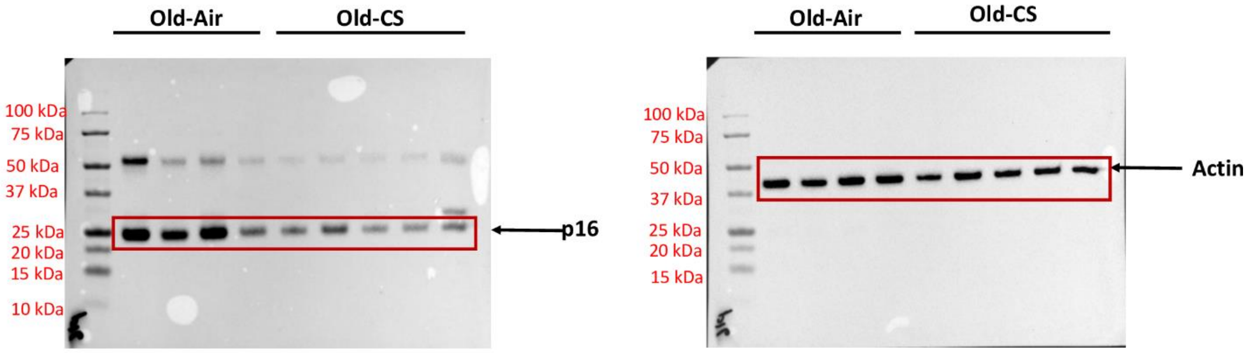

This shows the original uncut blots for p16 and β-actin as shown in Figure 4b of the manuscript

Uncut Original Blots

<u>Full unedited gel for Figure 5a</u>

p53 and respective β-Actin

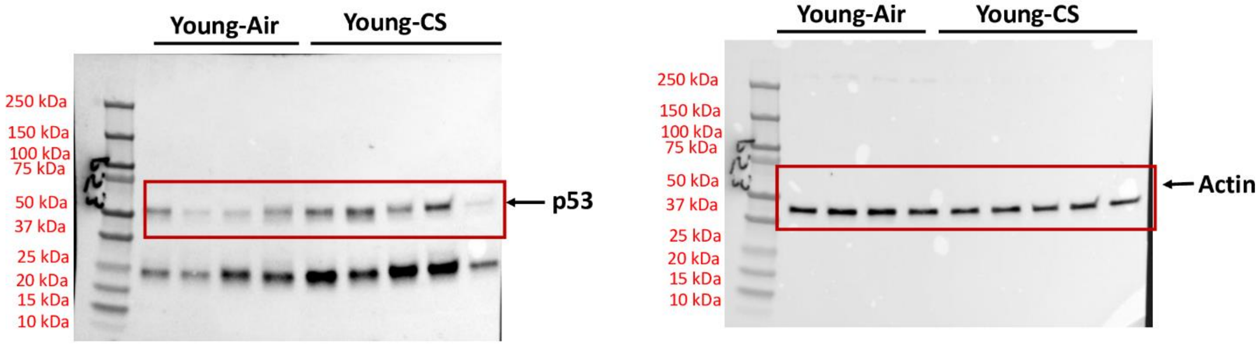

This shows the original uncut blots for p53 and β-actin as shown in Figure 6a of the manuscript

Uncut Original Blots

<u>Full unedited gel for Figure 5a</u>

p53 and respective β-Actin

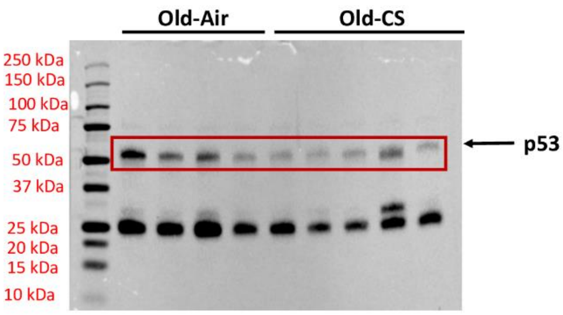

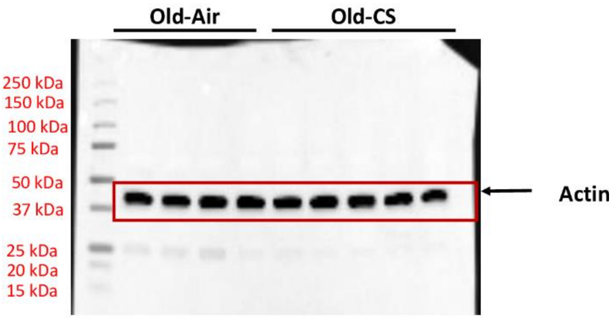

This shows the original uncut blots for p53 and β-actin as shown in Figure 6a of the manuscript

Uncut Original Blots

<u>Full unedited gel for Figure 6</u>

MMP9 and respective β-Actin

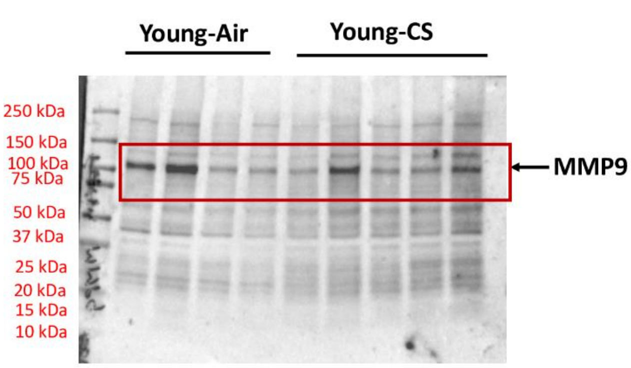

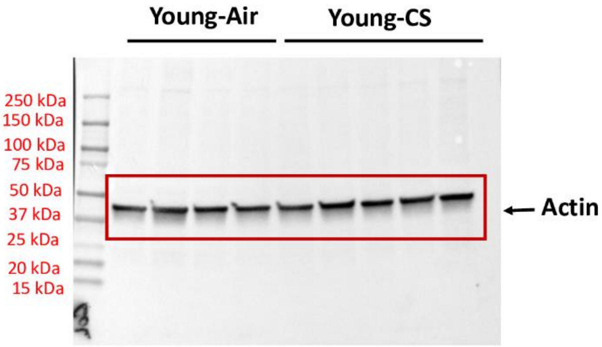

This shows the original uncut blots for MMP9 and β-actin as shown in Figure 5a of the manuscript

Uncut Original Blots

<u>Full unedited gel for Figure 6</u>

MMP9 and respective β-Actin

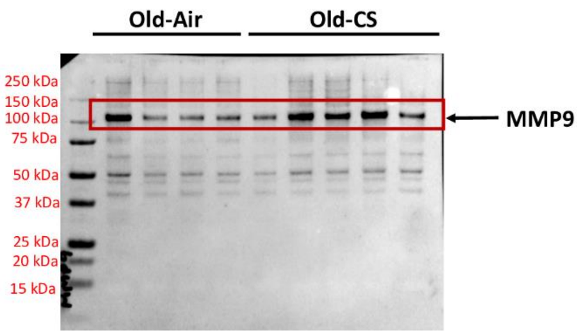

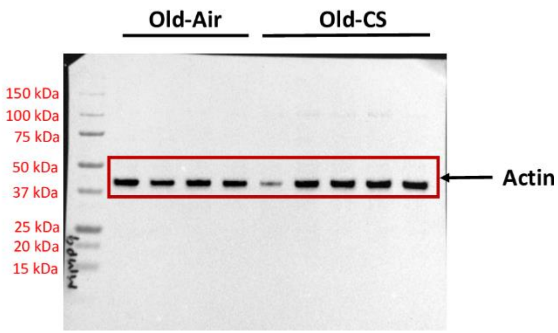

This shows the original uncut blots for MMP9 and β-actin as shown in Figure 5a of the manuscript

Uncut Original Blots

<u>Full unedited gel for Figure 6</u>
MMP12 and respective β-Actin

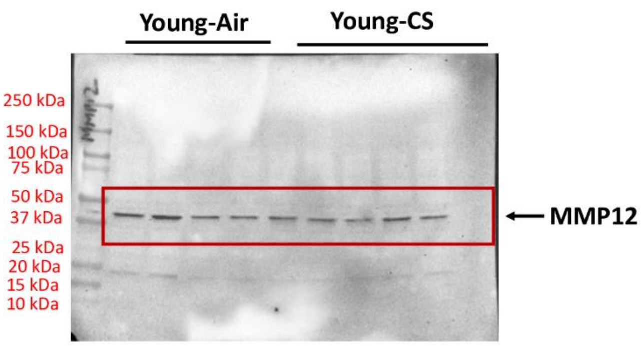

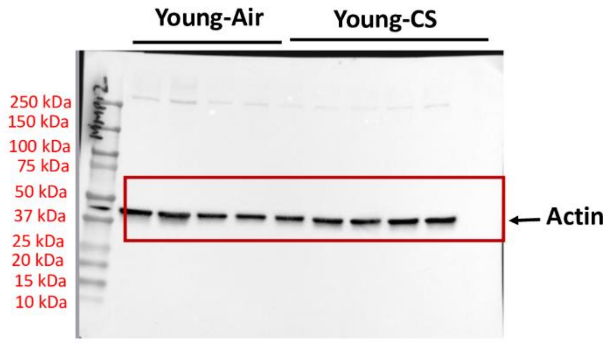

This shows the original uncut blots for MMP12 and β-actin as shown in Figure 5a of the manuscript

Uncut Original Blots

<u>Full unedited gel for Figure 6</u>
MMP12 and respective β-Actin

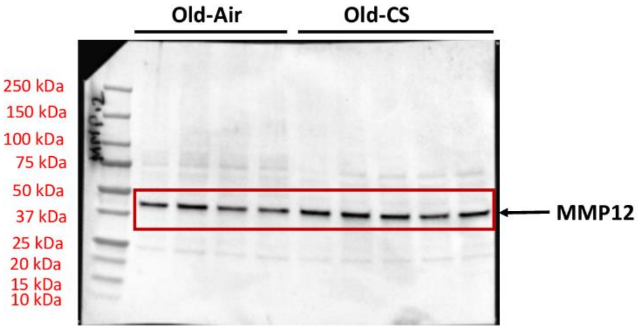

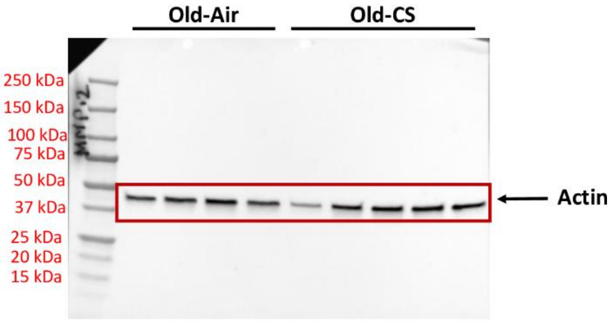

This shows the original uncut blots for MMP12 and β-actin as shown in Figure 5a of the manuscript

Uncut Original Blots

<u>Full unedited gel for Figure 6</u>
PAI-1 and respective β-Actin

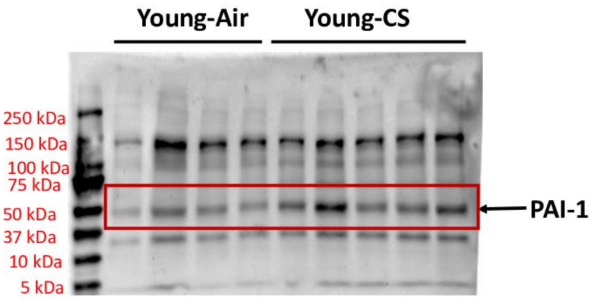

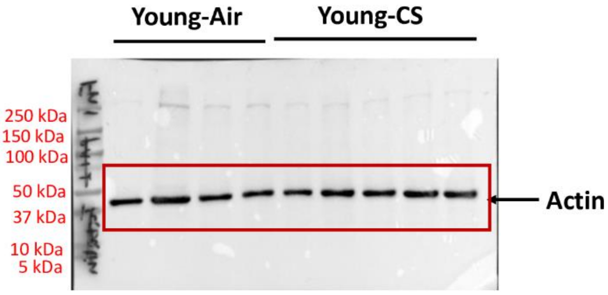

This shows the original uncut blots for PAI-1 and β-actin as shown in Figure 5a of the manuscript

Uncut Original Blots

Full unedited gel for Figure 6

PAI-1 and respective β-Actin

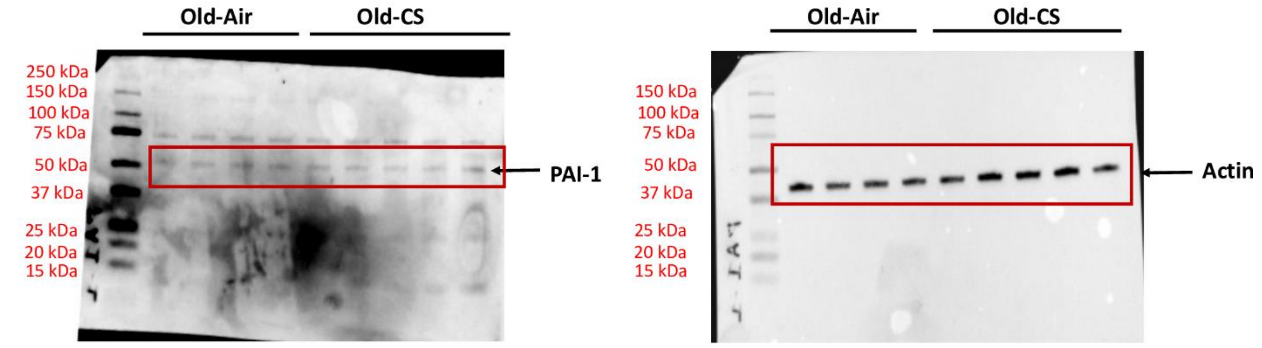

This shows the original uncut blots for PAI-1 and β-actin as shown in Figure 5a of the manuscript

Uncut Original Blots

Full unedited gel for Figure 6

Fn-1 and respective β-Actin

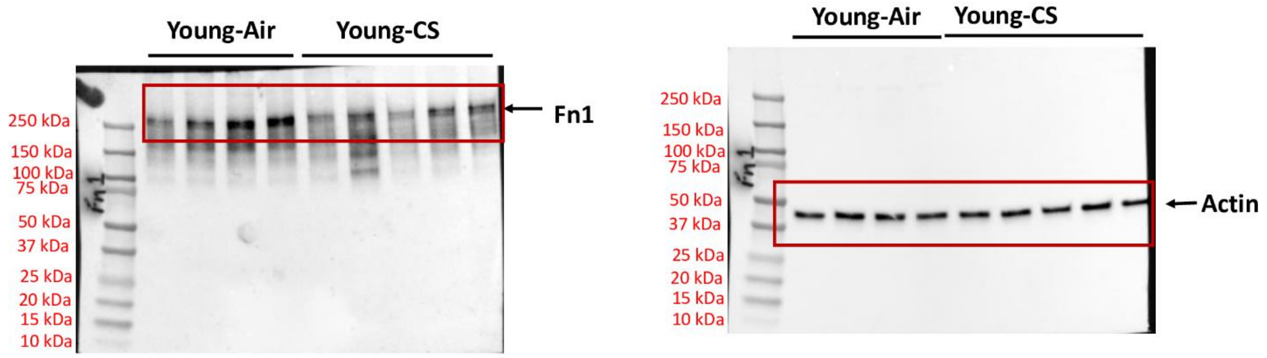

This shows the original uncut blots for Fn-1 and β-actin as shown in Figure 5a of the manuscript

Uncut Original Blots

Full unedited gel for Figure 6

Fn-1 and respective β-Actin

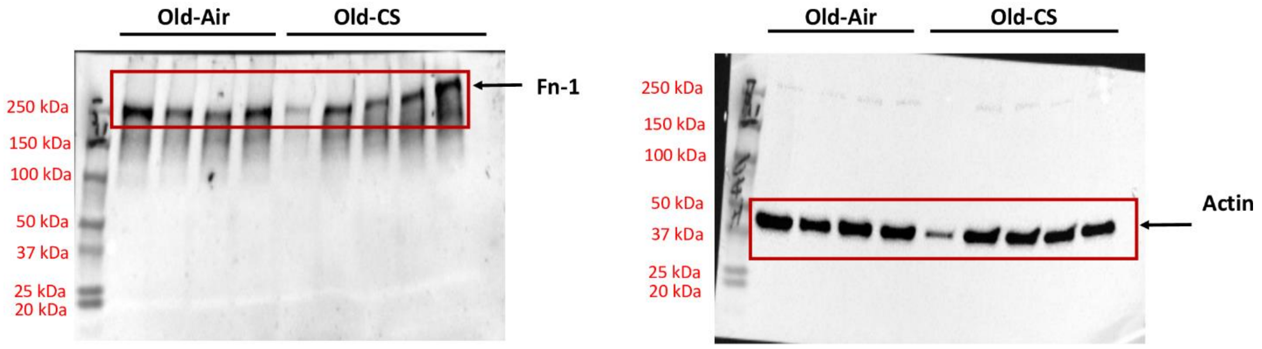

This shows the original uncut blots for Fn-1 and β-actin as shown in Figure 5a of the manuscript
